# Adoptive cell therapy against tumor immune evasion: mechanisms, innovations, and future directions

**DOI:** 10.3389/fonc.2025.1530541

**Published:** 2025-02-28

**Authors:** Liqin Ruan, Lu Wang

**Affiliations:** ^1^ Department of Hepatobiliary Surgery, JiuJiang City Key Laboratory of Cell Therapy, JiuJiang No.1 People’s Hospital, Jiujiang, Jiangxi, China; ^2^ Department of Oncology, JiuJiang City Key Laboratory of Cell Therapy, JiuJiang No.1 People’s Hospital, Jiujiang, Jiangxi, China

**Keywords:** adoptive cell therapy, tumor microenvironment, immune evasion, cancer mechanisms, personalized treatment

## Abstract

Tumors employ a range of strategies to evade detection and eradication by the host’s immune system. These include downregulating antigen expression, altering antigen presentation processes, and inhibiting immune checkpoint pathways. etc. Adoptive Cell Therapy (ACT) represents a strategy that boosts anti-tumor immunity. This is achieved by amplifying or genetically engineering immune cells, which are either sourced from the patient or a donor, in a laboratory setting. Subsequently, these cells are reintroduced into the patient to bolster their immune response against cancer. ACT has successfully restored anti-tumor immune responses by amplifying the activity of T cells from patients or donors. This review focuses on the mechanisms underlying tumor escape, including alterations in tumor cell antigens, the immunosuppressive tumor microenvironment (TME), and modulation of immune checkpoint pathways. It further explores how ACT can avddress these factors to enhance therapeutic efficacy. Additionally, the review discusses the application of gene-editing technologies (such as CRISPR) in ACT, highlighting their potential to strengthen the anti-tumor capabilities of T cells. Looking forward, the personalized design of ACT, combined with immune checkpoint inhibitors and targeted therapies, is expected to significantly improve treatment outcomes, positioning this approach as a key strategy in the field of cancer immunotherapy.

## 1 Background

Tumor immune escape refers to the process by which tumor cells evade recognition and elimination by the immune system through various mechanisms, allowing them to survive and proliferate within the body. This phenomenon is recognized as a key driver of tumorigenesis, progression, and recurrence (1). Tumors evade immune surveillance through various mechanisms, including downregulating antigen presentation, inhibiting T cell activity, and altering the function of immune cells in the tumor microenvironment (TME) (2). Notably, the activation of immune checkpoint pathways, such as PD-1/PD-L1 and CTLA-4, enables tumor cells to suppress T cell-mediated anti-tumor responses, thereby supporting their survival (3, 4). In recent years, in-depth research on immune escape mechanisms has provided important basis for the development of immunotherapies targeting these pathways (5, 6).

**Figure 1:**
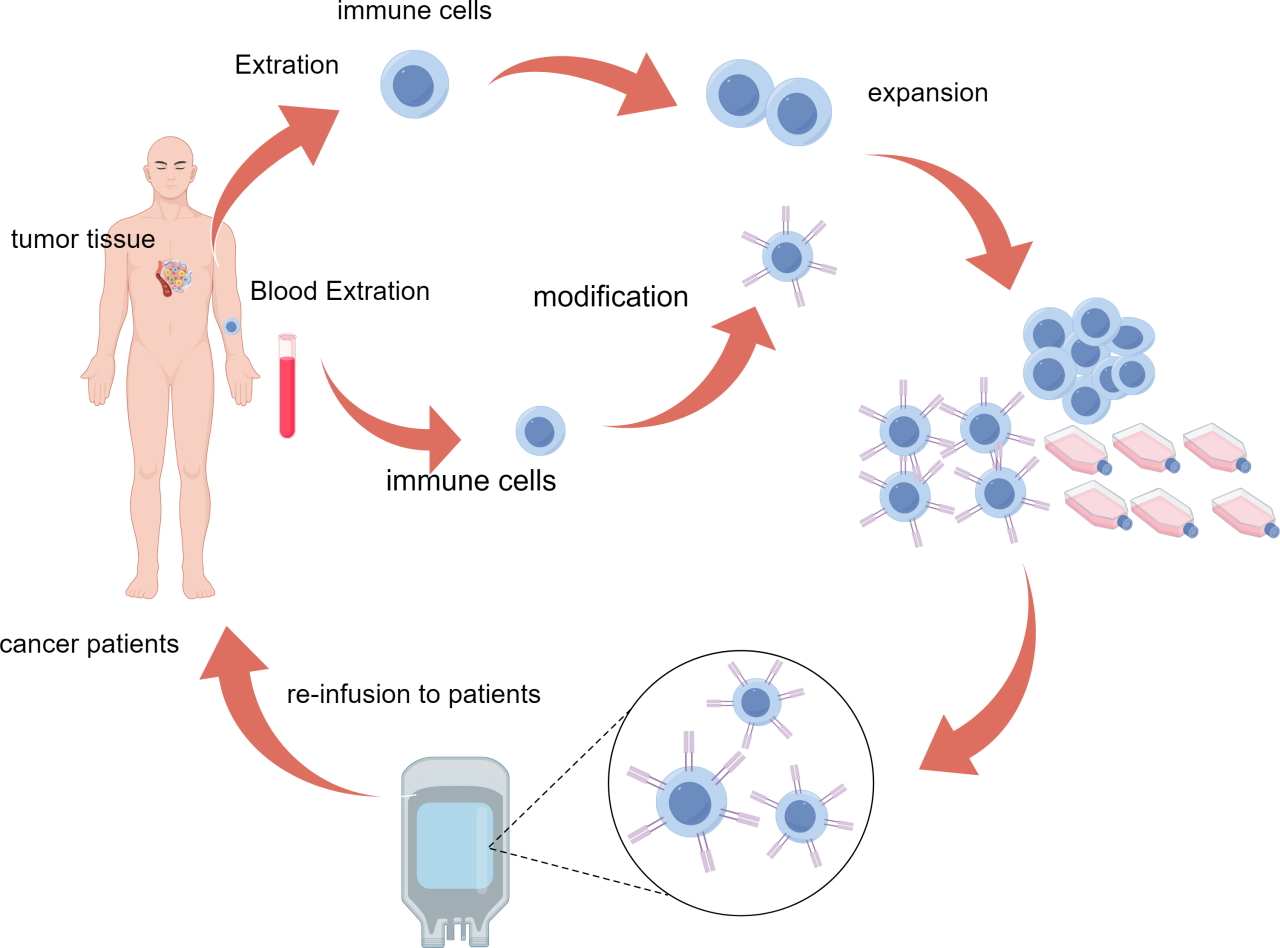
Schematic representation of the ACT process. Immune cells are isolated from patient tumor tissue or blood, genetically modified for enhanced tumor targeting, expanded ex vivo, and reinfused to attack cancer cells in the patient. ACT, adoptive cell therapy.

Adoptive cell therapy(ACT) refers to a treatment method that enhances the anti-tumor activity of a patient’s own or donor’s immune cells through *in vitro* expansion or genetic modification (7) (**Figure 1**). As an innovative cancer immunotherapy, ACT can effectively overcome tumor immune evasion and enhance the immune system’s ability to fight against tumors, showing a broad prospect for clinical application (8–10). ACT is currently classified primarily based on different anti-tumor mechanisms, including Tumor-infiltrating lymphocyte (TIL) therapy (11), Chimeric Antigen Receptor T cell (CAR-T) therapy (12), engineered T cell receptor (TCR-T) cell therapy (13) and Cytokine-Induced Killer cells(CIK) therapy (14), etc. (**Table 1**). In cancer treatment, ACT has become a key component of immunotherapy due to its high targeting and strong anti-tumor effects, especially showing a broad application prospect in the treatment of recurrent and refractory tumors (8, 10). In recent years, the combination of ACT therapy with immune checkpoint inhibitors (ICIs) has also shown significant efficacy (4–6).

**Table 1:** Summary of current adoptive cell therapies in cancer treatment.

Type	Cell source	Mechanism	Response Characteristic	Recent Clinical Trial
TIL (11)	Isolated from tumor tissue	TIL cells exert their anti-tumor effect by recognizing and killing tumor cells, activating immune responses, resisting tumor-induced immune suppression, and providing long-term immune memory.	adaptive immune cell	Creelan et al. (15) Huang et al. (16) Kristensen et al. (17) Rohaan et al. (18) Saberzadeh et al. (19) van den Berg et al. (20)
CAR-T (12)	PBMC, iPSC, UCB	T cells are engineered to express CARs that target tumor-specific antigens.	adaptive immune cell	Mailankody et al. (21) Majzner et al. (22) Narayan et al. (23) Qi et al. (24) Zhang et al. (25)
CAR- NK (26, 27)	PBMC, iPSC, UCB	NK cells are expanded *in vitro* or genetically modified to target tumors without MHC restriction	innate immune cell	Liu et al. (28) Marin et al. (29)
CAR-M (30)	PBMC, iPSC, hESC, UCB, hPSC, BM, cell lines	CAR-M cells enhance anti-tumor immunity by phagocytosing tumor cells, remodeling the tumor microenvironment, and promoting T-cell infiltration.	innate immune cell	NCT04660929(Active, not recruiting) NCT06224738(Not yet recruiting) NCT05007379(Unknown status)
CAR NKT (31)	PBMC, iPSC	CAR-NKT cells directly kill tumor cells by recognizing tumor antigens and release cytokines that activate other immune cells, enhancing the overall anti-tumor immune response with minimal side effects.	innate immune cell	Heczeyet al. (32) NCT06394622(recruiting) NCT06182735(recruiting) NCT03294954(recruiting) NCT03774654(recruiting)
CAR-γδT (33)	PBMC, iPSC	CAR-γδ T cells recognize and kill tumor cells through their unique TCR and CAR, while also activating immune responses to enhance anti-tumor effects.	innate immune cell	NCT06196294(Recruiting) NCT06196294(Recruiting) NCT06106893(Recruiting) NCT05388305(Unknown status) NCT04796441(Unknown status) NCT02656147(Unknown status) NCT04702841(Unknown status)
TCR-T (13)	PBMC, iPSC	TRT cells are genetically engineered to express TCRs that target specific tumor antigens presented by MHC	adaptive immune cell	Bear et al. (34) Krakow et al. (35) Ma et al. (36) Wermke et al. (37)
NRT (38)	Isolated from tumor tissue	NRTs target unique tumor-specific antigens, enabling precise anti-tumor activity with reduced off-target effects, while overcoming tumor microenvironment suppression to enhance therapeutic efficacy.	adaptive immune cell	Holm et al. (39) Kristensen et al. (17) Parkhurst et al. (40) Zacharakis et al. (41)
CIK (14)	PBMC	CIK cells are expanded *in vitro* using cytokines and are MHC-independent killers of tumor cells.	adaptive immune cell and innate immune cell	Li et al. (42) Ma et al. (43) Wang et al. (44) Zhang et al. (45)
DC-CIK (46)	PBMC	DC-CIK therapy combines dendritic cells' antigen-presenting role with the cytotoxic activity of cytokine-induced killer cells to activate and enhance immune responses against tumors.	innate immune cell	Jiang et al. (47) Wang et al. (48) Yang et al. (49) Zhan et al. (50) Zhao et al. (51)
CIML-NK (52)	PBMC, UCB, cell lines	CIML-NK cells are preconditioned with cytokines to acquire enhanced memory-like functions, can quickly produce high levels of IFNγ and cytotoxic responses when encountering tumor cells.	innate immune cell	Bednarski (53) Ciurea (54) Romee (55) NCT02782546(Recruiting) NCT05580601(Recruiting) NCT03068819(Recruiting) NCT06321484(Recruiting) NCT06138587(Recruiting)

M, macrophage; NK, natural killer; NKT, natural killer T; NRT, Neoantigen-Reactive T; CIK, Cytokine-Induced Killer Cell; DC, Dendritic Cells; CIML, Cytokine-Induced Memory-Like; PBMC, peripheral blood mononuclear cell; iPSC, induced pluripotent stem cell; UCB, umbilical cord blood; BM, bone marrow; Clinical trial data used in the table are from https://clinicaltrials.gov/, updated on November 4, 2024.

ACT effectively overcomes tumor immune evasion through various mechanisms, enhancing the immune system’s ability to recognize and eliminate tumors (56). ACT can enhance the recognition of tumor antigens by *in vitro* expansion of specific T cells or modification of immune cells (such as CAR-T cells),especially for the downregulation of antigen expression by tumor cells (12, 57, 58). Additionally, ACT can improve the immunosuppressive state in the TME. For instance, CIK cells have MHC-unrestricted killing characteristics and can counteract the negative effects of immunosuppressive cells (e.g., Treg cells, MDSC) in the TME (59–61). By targeting tumor-associated antigens and activating effector T cells, ACT can overcome challenges of immune checkpoint inhibition, such as the PD-1/PD-L1 pathway (4, 10). Furthermore, the combination of ACT with immune checkpoint inhibitors has shown potential to enhance T cell activity and reduce tumor drug resistance (5, 6). Through these innovative mechanisms, ACT not only increases the recognition rate of tumors but also significantly enhances the immune system’s cytotoxic effect on tumor cells (62, 63).

## 2 Overview of tumor immune evasion mechanisms

**Figure 2:**
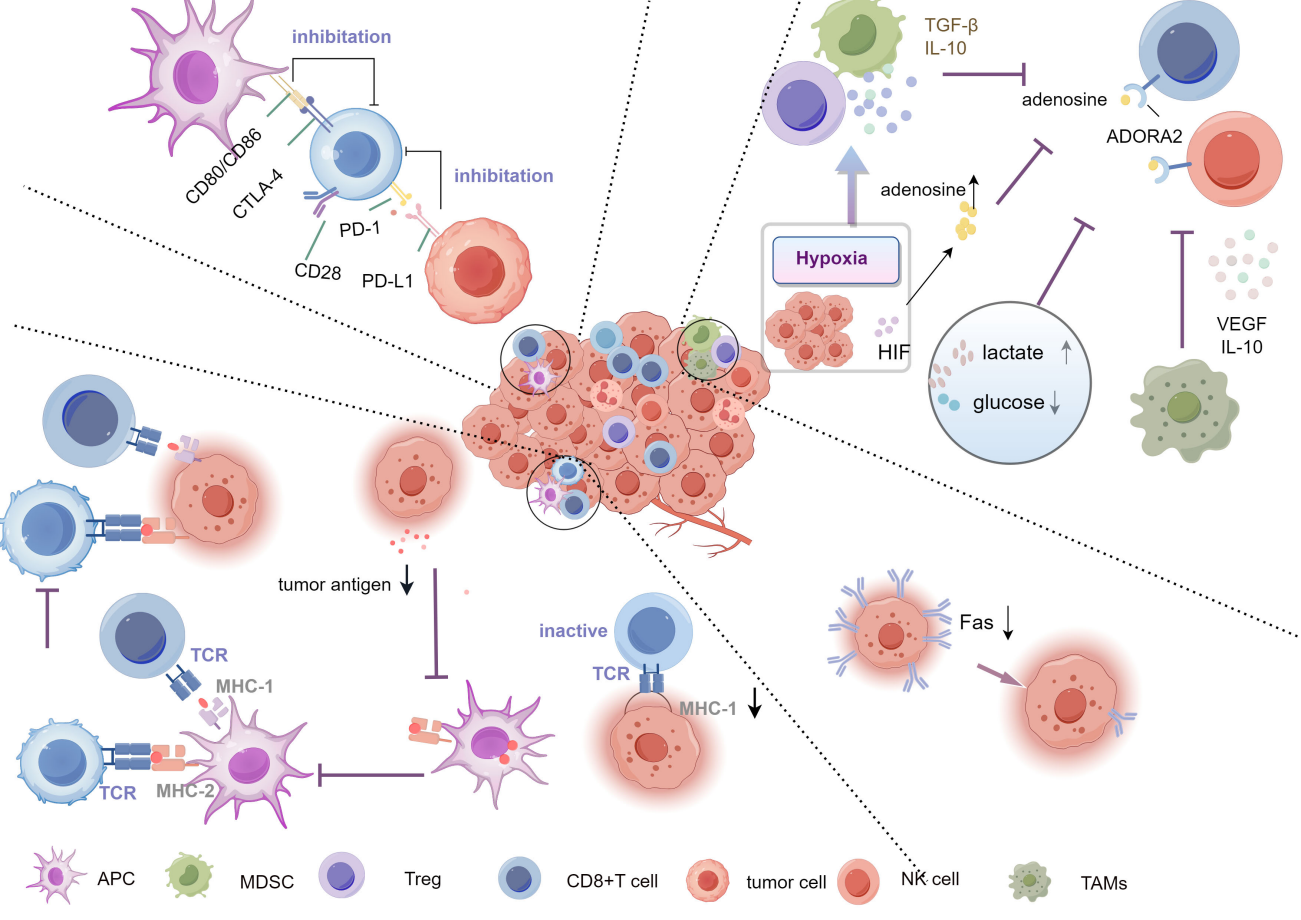
Mechanisms of tumor immune evasion. The Key mechanisms include downregulation of antigen expression or alteration of antigen presentation, preventing effective immune recognition, inhibiting T cell activation via checkpoints (PD-1/PD-L1, CTLA-4), and creating an immunosuppressive microenvironment rich in TGF-β,IL-10, and adenosine. Hypoxia and metabolic changes (high lactate, low glucose) further support evasion, aided by Tregs, TAMs, and MDSCs. Tregs, Regulatory T cells; TAMs, tumor-associated macrophages; MDSCs, myeloid-derived suppressor cells.

The causes of tumor immune evasion are complex and diverse, making it one of the key factors in tumor growth and metastasis. Firstly, tumor cells evade recognition and attack by T cells by downregulating or altering the expression of major histocompatibility complex (MHC) molecules (61–66). Secondly, the TME is rich in immunosuppressive cells, such as regulatory T cells (Tregs) and myeloid-derived suppressor cells (MDSCs), which weaken the function of effector T cells by secreting inhibitory cytokines (e.g., TGF-β, IL-10) (4, 67, 68). Moreover, tumor cells bind to PD-1 on the surface of T cells by expressing immune checkpoint molecules such as PD-L1, leading to T cell exhaustion and further suppression of anti-tumor immune responses (5, 6, 69). The synergistic effect of these evasion mechanisms enables tumor cells to endure and proliferate within the host (**Figure 2**).

### 2.1 Downregulation of antigen expression

Tumor cells evade immune system recognition by reducing antigen expression or altering the antigen presentation process, which is one of the key mechanisms of tumor immune evasion (59, 62, 64). Normally, cells present antigens through MHC molecules, enabling T cells to recognize and kill abnormal cells. The downregulation of tumor neoantigens can be induced through various pathways, such as copy number loss, transcriptional repression, epigenetic silencing, and post-translational mechanisms. These processes affect tumor antigen presentation (70). However, many tumor cells alter this process through various pathways, avoiding immune surveillance (71, 72). Firstly, tumor cells can downregulate the expression of MHC class I (MHC-I) molecules, which are key elements in presenting intracellular protein fragments (including tumor antigens) to CD8^+^ T cells (73). If the expression of MHC-I molecules is reduced, tumor antigens will not be effectively presented, and CD8^+^T cells will fail to recognize and attack these tumor cells (3, 62). For instance, downregulation or loss of MHC-I is frequently observed in tumors such as melanoma and lung cancer (74, 75). Moreover, tumor cells can also alter the antigen processing machinery, inhibiting the loading of tumor antigens onto MHC molecules, thereby further reducing the likelihood of immune recognition (76, 77). Additionally, accessory proteins involved in the antigen presentation process may also be modulated. For example, tumor cells can reduce the function of key molecules in the antigen processing machinery, such as the proteasome and TAP transporters, further hindering the presentation of antigens on MHC-I (78). Other proteins in the antigen presentation process, such as β2-microglobulin, are also often downregulated in tumor cells, leading to unstable expression of MHC molecules on the cell surface and further reducing the chances of tumor cells being recognized by the immune system (79).

Furthermore, tumor cells can also produce incompletely or misfolded proteins through antigen mutation, further avoiding T cell recognition (80). In some instances, tumor cells might even cease to express tumor-specific antigens, thereby fundamentally evading immune system assaults (81). Such antigen mutation and loss are common in advanced tumors and are closely related to tumor invasiveness and metastasis (82). This immune evasion mechanism, which reduces antigen expression or alters the antigen presentation process, allows tumor cells to continue growing and expanding under immune surveillance. Strategies to overcome this evasion mechanism have become an important research direction in current cancer immunotherapy. For example, restoring the expression of MHC-I molecules, enhancing antigen presentation capabilities, and ACT that can recognize atypical antigens have shown some efficacy in clinical studies (83, 84).

### 2.2 Immunosuppressive TME

The immunosuppressive characteristics of the TME play a crucial role in tumor immune evasion (85–87). The TME not only consists of tumor cells, but also includes immune cells, stromal cells, blood vessels, as well as various signaling molecules, which together constitute a complex immunosuppressive network (88). Among them, Tregs and MDSCs in the TME are major participants in immunosuppression. They suppress the activity of effector T cells and natural killer (NK) cells by secreting inhibitory cytokines such as TGF-β and IL-10, weakening the body’s immune response to the tumor (89, 90).

Additionally, tumor-associated macrophages (TAMs) present in the TME also promote immune evasion. TAMs often exhibit an M2 phenotype, which is characterized by their roles in promoting tissue repair and suppressing inflammatory responses. M2-type TAMs facilitate tumor angiogenesis and immunosuppression and inhibit the function of T cells by secreting factors such as VEGF and IL-10 (91, 92). Studies have shown that the density of TAMs at tumor sites is closely associated with tumor progression and poor prognosis (93). Hypoxia is another important characteristic of the TME. Rapid tumor proliferation leads to local hypoxia, activating HIF, which promote the expression of immunosuppressive molecules (94). The hypoxic environment also induces the generation of adenosine, which inhibits the function of T cells and NK cells through the A2A receptor (95). Furthermore, hypoxic conditions can enhance the immunosuppressive effects of Tregs and MDSCs, further weakening the anti-tumor immune response (96).

Metabolic inhibition is also an important mechanism of immune evasion in the TME. Tumor cells in the TME consume large amounts of nutrients such as glucose and glutamine, leading to restricted metabolic activity of effector T cells and preventing them from functioning properly (97). Additionally, the accumulation of lactic acid in the TME also suppresses the proliferation and cytotoxicity of T cells by acidifying the environment (98).

Overall, the TME employs various immunosuppressive mechanisms, including the regulation of immune cell activity, hypoxia, and metabolic changes, to help tumors escape surveillance. The interaction of these mechanisms allows tumors to continue growing and spreading under immune pressure. Therefore, therapies targeting the immunosuppressive TME, such as targeting Tregs, TAMs, or restoring T cell metabolic activity, have become important directions for improving the efficacy of tumor immunotherapy (99).

### 2.3 Escape through immune checkpoint pathways

Immune checkpoint pathways play a crucial role in tumor immune evasion, allowing tumor cells to evade attacks from the host immune system by suppressing T cell functions. Immune checkpoints are important mechanisms that regulate the intensity and duration of immune responses, designed to prevent an overactive immune system from causing autoimmunity (100, 101). By activating these pathways, tumor cells weaken the anti-tumor activity of T cells, thereby helping the survival and spread of tumor cells (100–102). One of the most widely studied immune checkpoint pathways is the PD-1/PD-L1 (programmed death protein-1) pathway. PD-1 is an inhibitory receptor expressed on the surface of T cells, and when it binds to its ligand PD-L1, the PD-1 pathway inhibits T cell proliferation, cytokine secretion, and cytotoxic activity. Many tumor cells highly express PD-L1, which binds to PD-1 on T cells, preventing T cells from attacking tumor cells (62, 103, 104). Studies have shown that overexpression of PD-L1 in various tumors, such as melanoma, lung cancer, and liver cancer, is associated with tumor progression and poor prognosis (105).

In addition to the PD-1/PD-L1 pathway, Cytotoxic T-Lymphocyte Associated Antigen-4 (CTLA-4)is another crucial immune checkpoint. CTLA-4 competitively binds to CD80 and CD86 with the T cell co-stimulatory molecule CD28, and its inhibitory effect on T cell activity is relatively early, primarily occurring in the lymph nodes (6, 106). Beyond directly affecting T cell function, immune checkpoint pathway also further suppresses the anti-tumor immune response by regulating immune suppressive cells in the TME, such as Tregs and MDSCs. Tregs and MDSCs enhance the tumor’s immune evasion capabilities by highly expressing checkpoint molecules like PD-L1 (107, 108). Moreover, tumor cells can also adapt to immunotherapy pressure by inducing the expression of checkpoint molecules, rendering traditional immunotherapy ineffective (109).

In recent years, therapies targeting immune checkpoint pathways, particularly inhibitors of PD-1/PD-L1 and CTLA-4, have become significant breakthroughs in cancer treatment. By blocking these inhibitory pathways, immune checkpoint inhibitors can restore the anti-tumor functions of T cells (10, 110, 111). However, despite the significant clinical efficacy of immune checkpoint inhibitors, some patients still do not respond to treatment or eventually develop resistance, which may be related to the tumor’s ability to evade immune attacks through multiple escape mechanisms (2, 80). Therefore, researching how to overcome the evasion of immune checkpoint pathways is a key direction for improving the effectiveness of cancer immunotherapy in the future.

### 2.4 Other reasons

The mechanisms of tumor immune evasion are diverse. In addition to downregulation of MHC molecules and escape through immune checkpoint pathways, the Fas/FasL pathway blockade is also common mean of immune evasion (112, 113). The Fas/FasL pathway is an important mechanism for regulating apoptosis; under normal conditions, the binding of the Fas receptor to its ligand(FasL) induces apoptosis (114). However, many tumor cells evade apoptosis signals mediated by T cells and B cells by reducing the expression of Fas receptors or altering the function of FasL, thereby achieving immune evasion (3, 62, 115). For instance, research has found that various solid tumors, including melanoma, exhibit dysregulation of the Fas/FasL pathway (116–118). Immune escape mechanisms governed by mutated NOTCH in mature B-cell malignancies, mediated by increased PD-L1 expression and downregulation of MHC class II genes (119). These complex mechanisms allow tumors to evade immune attacks through multiple pathways, suggesting that interventions targeting the Fas/FasL pathway could become new directions for future tumor immunotherapy.

## 3 The foundation and development of ACT

ACT is a treatment method based on T cells from patients or donors, which enhances their anti-tumor activity through *in vitro* expansion or genetic modification, and then reinfuses the expanded T cells or CIK cells back into the patient’s body to strengthen their anti-tumor activity, avoiding the weakening effects of inhibitory factors in the tumor microenvironment (120). The origin of ACT can be traced back to the 1980s when Rosenberg and colleagues first reported the application of TIL therapy in the treatment of melanoma (121). Since then, ACT has gone through several stages of development. Particularly with the advancement of genetic engineering technology, CAR-T cell therapy and TCR-T cell therapy have become the representatives of modern ACTs (6, 10, 122).

ACT works by overcoming multiple immune evasion mechanisms of tumors. TIL therapy amplifies T cells isolated from tumors, enhancing their ability to recognize and attack tumor cells (99). CAR-T therapy, on the other hand, enables T cells to recognize specific antigens on the surface of tumor cells through genetic modification, thus circumventing immune evasion caused by the downregulation of MHC-I. Moreover, the *in vitro* modification of CAR-T and TCR-T cells can make them tolerant to immune checkpoint molecules, thereby avoiding inhibition through the PD-1/PD-L1 pathway (2, 5, 10, 122–124).

## 4 Mechanisms of overcoming tumor immune evasion in ACT

### 4.1 Specific antigen recognition

**Figure 3:**
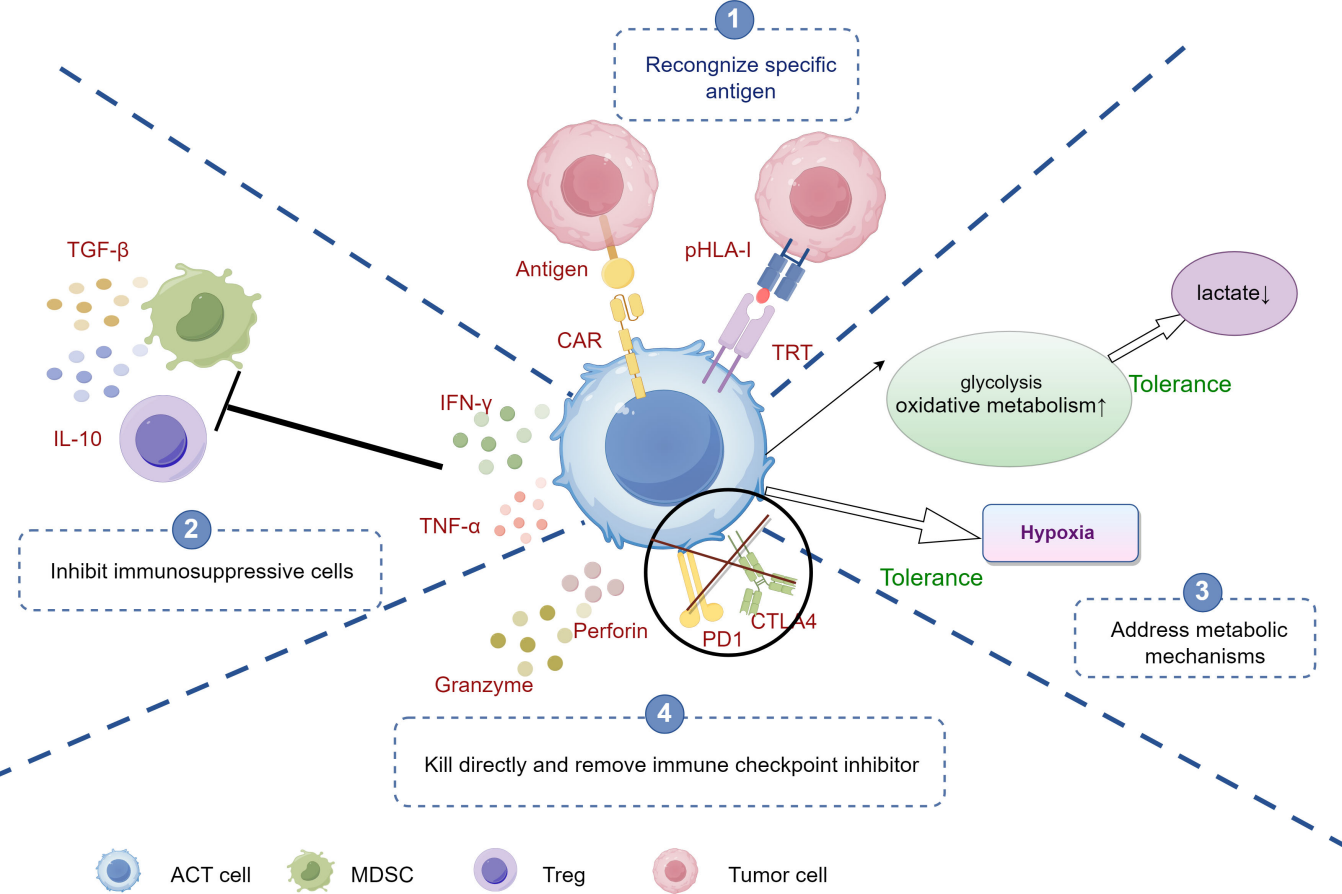
The mechanisms of ACT against tumor immune evasion. ①Antigen Recognition: CAR-T cells recognize specific antigens on tumor cells, initiating targeted attack. ②Inhibition of Immunosuppressive cells: CAR-T cells counteract immunosuppressive cells (e.g., Tregs, MDSCs) by blocking signals, enhancing immune response. ③Addressing Metabolic Barriers: CAR-T cells adapt to the tumor microenvironment by increasing glycolysis and oxidative metabolism, enabling function under hypoxia and low lactate conditions. ④Direct Killing and Immune Checkpoint Inhibition: CAR-T cells release cytotoxic molecules (e.g., perforin, granzyme) to kill tumor cells and target immune checkpoints (e.g., PD-1, CTLA-4) to overcome tumor-induced tolerance. ACT, adoptive cell therapy; CAR-T, Chimeric Antigen Receptor T-Cell; Tregs, Regulatory T cells; TAMs, tumor-associated macrophages; MDSCs, myeloid-derived suppressor cells; Tregs, Regulatory T cells; MDSCs, myeloid-derived suppressor cells.

Specific antigen recognition is one of the core mechanisms by which ACT enhances T cell anti-tumor activity and effectively overcomes the issue of tumor immune evasion (6). Tumor cells often escape immune surveillance by reducing their antigen exposure or downregulating MHC (**Figure 3**). However, in ACT, specific T cells can recognize tumor-associated antigens and restore their anti-tumor function through genome editing technologies such as CRISPR. Even when MHC expression is reduced, by modifying T cells *in vitro*, their anti-tumor activity is enhanced, avoiding the interference of tumor evasion mechanisms (10, 12, 63). Particularly, CAR-T therapy uses genetic engineering to equip T cells with specific antigen receptors that can recognize specific antigens on the tumor surface, allowing T cells to bypass these evasion mechanisms and precisely identify tumor cells, avoiding the MHC-dependent recognition process (10, 12). For instance, CAR-T cells can recognize the highly expressed CD19 antigen in hematological tumors, effectively attacking tumor cells regardless of MHC downregulation (125). This design overcomes the tumor’s MHC downregulation evasion strategy, significantly enhancing the anti-tumor effects of T cells.

TCR-T cells are engineered to introduce specific TCRs that enable them to recognize tumor endogenous antigens presented by MHC molecules (13). Although TCR-T cells rely on MHC, they can recognize a variety of tumor endogenous and specific mutational antigens, making them applicable to a broader range of tumor types. For instance, TCR-T cells can recognize the MART-1 antigen in melanoma, effectively activating T cells to kill tumors. Scientists have also genetically modified TCR-T cells to maintain a higher affinity for tumor antigens, further enhancing their recognition and killing capabilities.

To address tumor heterogeneity and antigen escape, bispecific and multispecific T cell technologies are emerging. By designing T cells capable of recognizing two or more tumor antigens, ACT increases the therapeutic coverage and prevents tumors from escaping attack through antigen loss. For example, bispecific CAR-T cells can simultaneously recognize CD19 and CD22, improving anti-tumor efficacy and reducing the risk of relapse (126).

With the advancement of genomics, personalized T cell therapies targeting patient-specific neoantigens have gradually been applied. These neoantigens are generated by mutations and do not exist in normal cells, making them ideal therapeutic targets. By identifying neoantigens through genetic sequencing and designing exclusive TCRs, personalized T cells can be generated that specifically target the patient’s tumor, effectively addressing refractory tumors.

### 4.2 Overcoming immune checkpoint inhibition

In the TME, tumor cells often suppress the activity of T cells through immune checkpoint pathways, such as PD-1/PD-L1 and CTLA-4, thereby evading immune surveillance. When PD-L1 binds to PD-1 on T cells, it inhibits T cell proliferation and cytotoxicity, ultimately leading to a state of T cell exhaustion, where they cannot recognize and attack tumor cells normally (127). To bypass this inhibitory mechanism, researchers have used gene-editing technologies (such as CRISPR-Cas9) to knock out the PD-1 gene on the surface of T cells, thereby blocking the inhibitory signals of the PD-1/PD-L1 pathway and allowing T cells to remain active in the TME. Studies have shown that PD-1 knockout (KO) T cells exhibit stronger proliferative capacity and anti-tumor activity in both *in vitro* and *in vivo* experiments, avoiding negative feedback regulation by immune checkpoints *in vivo* (2, 10). Particularly, CAR-T cells has been genetically modified to make T cells no longer dependent on MHC molecules recognition of tumors and capable of resisting inhibition by the PD-1 and CTLA-4 pathways (12, 63).

Immune checkpoint inhibitors enhance anti-tumor immune responses by removing inhibitory signals in the immune system, but their clinical application still faces significant bottlenecks, including limited efficacy, uncertainty of predictive biomarkers, immune-related adverse events, and high treatment costs (128). ACT can be used in combination with immune checkpoint inhibitors (such as PD-1, PD-L1, or CTLA-4 inhibitors) to relieve the suppression of T cell functions and further enhance the anti-tumor immune response (5, 99). Combined treatment strategy (chemotherapy, radiotherapy, immune checkpoint blockers) has shown significant efficacy in various types of tumors, especially for patients with advanced and recurrent tumors. The combination of ACT and immune checkpoint inhibitors can significantly improve treatment response rates and patient survival (129, 130). Future research directions aim to further optimize this combined strategy to reduce side effects and improve long-term efficacy.

### 4.3 Improving immunosuppression in the tumor microenvironment

The TME is rich in immunosuppressive cells, such as Tregs, MDSCs, and TAMs, which weaken the anti-tumor functions of effector T cells by secreting immunosuppressive factors (such as TGF-β, IL-10) (131–135). ACT can reshape the TME and restore immune responses through various pathways.

Initially, adoptive T cell therapy can reverse the immunosuppressive state by secreting cytokines (such as IFN-γ, TNF-α) (10, 136, 137). These cytokines suppress the function of Tregs and MDSCs, reducing their immunosuppressive effects in the TME and restoring the activity of effector T cells and NK cells (63, 138). Likewise, ACT can directly reduce the number of these suppressive cells by clearing Tregs and MDSCs from the TME, promoting anti-tumor immune responses (139, 140).

In addition, the TME is often characterized by hypoxia, nutrient deprivation, and the accumulation of metabolic waste products such as lactate, which significantly suppress the activity of effector T cells (62, 97). ACT modifies T cells genetically to better adapt to these adverse metabolic conditions, thereby maintaining their anti-tumor functions (98). Genetically modified T cells can enhance their glycolytic or oxidative metabolism capabilities, allowing them to remain active in environments lacking glucose or with energy constraints (111, 141).

Tumor cells typically weaken the energy supply of effector T cells through metabolic suppression; however, CAR-T cells can be designed to tolerate nutrient-poor environments, thus preserving their cytotoxic functions (6). ACT can synergize with immune checkpoint blockade therapies to enhance the anti-tumor immune response. Immune checkpoint pathways, such as PD-1/PD-L1 and CTLA-4, are prevalent in the TME and are utilized by tumors to suppress T-cell activity (80). By combining ACT with immune checkpoint inhibitors (e.g., PD-1 and CTLA-4 inhibitors), these inhibitory signals within the TME can be blocked, further activating effector T cells (5, 99). In summary, ACT not only directly enhances the anti-tumor function of T cells but also modulates immunosuppressive mechanisms within the TME, reducing tumor immune evasion and improving the overall efficacy of cancer therapy.

### 4.4 Addressing metabolic and other immune evasion mechanisms

ACT has demonstrated significant potential in overcoming the metabolic suppression within the TME, particularly through genetic modifications that enhance T-cell adaptability. The TME is typically characterized by hypoxia, nutrient deprivation, and the accumulation of metabolic waste products, such as lactate, which severely inhibit the activity of effector T cells (5, 63). ACT leverages genetic modifications to equip T cells to better adapt to these adverse metabolic conditions, thereby sustaining their anti-tumor functionality.

First, hypoxia is a defining feature of the TME. While tumor cells adapt to low oxygen levels by activating the HIF pathway, effector T cells are often inhibited under such conditions (94). Research indicates that genetically modifying adoptive T cells to overexpress HIF-stabilizing proteins or enhance their metabolic activity can improve T-cell survival and function in hypoxic environments (98).

Second, glucose deprivation and lactate accumulation in the TME restrict the energy supply to T cells, suppressing their anti-tumor activity (97). Genetically modified T cells can be engineered to enhance their glycolytic or oxidative metabolic pathways, allowing them to maintain activity even in glucose-deprived or energy-limited environments (63, 99). Moreover, engineered T cells can be made resistant to the high levels of lactate in the TME, thereby minimizing the inhibitory effects of lactate on T-cell function (2, 142).

Lastly, adenosine accumulation in the TME inhibits T-cell function through the A2A receptor pathway (95). Genetically modifying T cells to resist adenosine signaling enables them to retain their cytotoxic activity in adenosine-rich TME (5, 96). These metabolic adaptation strategies not only enhance T-cell survival in the TME but also significantly improve their anti-tumor efficacy (6, 10).

## 5 Applications of adoptive cell therapy across different tumor types

ACT has shown significant therapeutic efficacy in multiple tumor types through enhancing T-cell immune responses to tumors, especially in overcoming tumor immune escape mechanisms. The core of ACT involves extracting T cells from the patient, expanding or genetically modifying them *in vitro* to enhance their anti-tumor potency, and then re-infusing them into the body to restore or boost the patient’s immune response (12). This treatment has demonstrated potent anti-tumor potential in clinical trials for various tumors, including melanoma, lung cancer, breast cancer, and lymphoma (15, 143–145) (**Table 2**).

**Table 2:** Clinical trials of ACT in tumors with published results.

Tumor Type	Clinical trial	Phase	ACT Type	N	Clinical response	AEs related to ACT
Melanoma	Morgan et al. (146)	I	TCR-T	17	2 PR (20-21 m); ORR: 11.8%	None
	Ten Ham et al. (18)	III	TIL (vs Ipi)	84	17 CR(20-21 m), 24 PR (4.2-13.1m); ORR:49 %	Fever(92%), Chills(84%),
	Bol KF et al. (147)	III	DC	99	Median RFS: 12.7 m	Flu like symptoms(41%), Pain injection site (34%), fatigue (33%)
Melanoma and colorectal cancer and sarcoma	Gargett et al. (148)	I	CAR-T	12	5 PR (NE); ORR: 41.7%	Rash (50%), fever (33%), diarrhea (33%) and anorexia (33%)
Lung Cancer	Zhou et al. (149)	IB	CIK+Chemo	34	2 CR(>20.5and >21m), 5 CMR(>4.5 to >24m), 21 PR (8.3m-NA); ORR:82.4 %	Anemia(67.6%), Leukopenia(67.6%), Nausea(64.7%)
	Creelan et al. (15)	I	TILs + Nivo	13	1 CR (>18 m), 2 PR (>12 to >23 m); ORR: 23%	Nausea (86%), skin rash (55%), diarrhea (55%), CRS (45%); total severe toxicity: 12.5%
	Zhang et al. (150)	I	CAR-T	9	1 PR (>13 m); ORR: 11.1%	Fever(77.8%), Chill(22.2%), Muscle weakness(22.2%)
Neuroblastoma	Heczey, A.et al (32)	I	CAR-NKT	12	1 CR (6 m), 3 PR (1-3.5 m); ORR: 33%	Neutropenia(100%), Leukopenia(91.7%), Lymphopenia(83.3%)
Breast Caner	Zacharakis et al. (41)	II	TILs+Pembro	6	1 CR(>66 m); 2PR(6-10m);ORR:50%	NE
Mesothelioma	Adusumilli et al. (151)	I	CAR-T+Pembro	23	2 PR(NE) ORR:8.7%	Fatigue (52%), Fever (52%), Pain (49%)
Recurrent/Refractory B cell non-Hodgkin lymphoma	Wang et al. (152)	I/II	CAR-T	11	7 CR(4.7-18.5m ) 2 PR(NE) ORR:90%	Fatigue ( 64 %) Anorexia ( 64%) Neutropenia (64%)
Relapsed/Refractory Multiple Myeloma	Jurgens et al. (153)	I	CAR-T	17	7 CR(3m-NE) 5 PR(1-24m) ORR:71%	Nail changes (65%), Rash (18%), Dysgeusia(18%) Neurotoxicity(18%)

AEs, adverse events; CR, complete responses; CMR, complete metabolic response; Chemo, Chemotherapy; CRS, cytokine release syndrome; Ipi, ipilimumab; m, months; NE, not specified; Nivo, nivolumab; ORR, objective response rate; PR, partial responses; Pembro, pembrolizumab; RFS, relapse free survival.

### 5.1 ACT in melanoma

Melanoma is one of the earliest cancers to receive adoptive cell therapy, with research focusing on TIL therapy (154). TIL therapy involves the amplification of T cells isolated from the patient’s tumor tissue, successfully overcoming the tumor’s immune escape through downregulation of antigen expression and immunosuppressive TME (4, 99). A key clinical trial indicated that about 50% of patients with advanced melanoma experienced significant tumor reduction after receiving TIL therapy, with some patients remaining recurrence-free in the long term (18). The antigen specificity of TILs enhances T cell recognition of tumor antigens, while the secretion of cytokines such as IFN-γ suppresses immunosuppressive cells (155). Additionally, studies have shown that the combination of TIL therapy with PD-1 or CTLA-4 inhibitors further enhances the therapeutic effect (156). Immune checkpoint inhibitors release the inhibitory state of T cells, allowing them to exert a stronger cytotoxic effect in the tumor microenvironment (63). This strategy effectively overcomes the immune escape of tumor cells with high PD-L1 expression.

### 5.2 ACT in lung cancer

Lung cancer, as a highly heterogeneous tumor, has a variety of immune escape mechanisms. Studies have shown that lung cancer cells often downregulate the expression of MHC-I, weakening antigen presentation capabilities and preventing T cell recognition (157). In a CAR-T therapy trial for lung cancer patients, CAR-T therapy was able to effectively recognize and kill tumor cells with insufficient MHC I expression, compensating for the limitations of traditional T cells that rely on MHC recognition (97, 98). Additionally, hypoxia is another characteristic of the lung cancer microenvironment, leading to immune escape. Genetically modified CAR-T cells, by increasing their adaptability to hypoxic conditions, can remain active even under these adverse conditions (95). This enhanced metabolic adaptability helps T cells function in a microenvironment with nutrient deprivation and accumulation of metabolic waste, overcoming the suppression of T cell function (96).

### 5.3 ACT in breast cancer

In the TME of breast cancer patients, the number of Tregs and MDSCs is significantly increased, suppressing the anti-tumor activity of effector T cells (5, 158). In a clinical trial of ACT for advanced breast cancer, researchers used CAR-T cells with enhanced antigen specificity through genetic modification (80). The results showed that CAR-T cells could effectively suppress the immunosuppressive effects of Tregs and MDSCs, restoring the activity of effector T cells (62).

Furthermore, the high expression of the immune checkpoint molecule PD-L1 in breast cancer cells also helps in immune evasion (159). *In vitro* model studies have shown that the combination of ACT with PD-L1 inhibitors produced a good anti-cancer response in autologous Patient-Derived Xenograft(PDX) models of advanced triple-negative breast cancer (160). This combined strategy not only enhances the recognition of tumors by CAR-T cells but also allows more T cells to function by releasing immune checkpoint inhibition (129, 130, 156, 160).

### 5.4 ACT in lymphoma

Lymphoma is another successful application of CAR-T therapy, especially in B-cell lymphoma (161). B-cell lymphoma cells often evade immune surveillance through immune escape mechanisms such as downregulating antigen expression or altering antigen presentation processes (119). In a clinical trial, the use of CD19-targeted CAR-T cell therapy for relapsed B-cell lymphoma achieved a high rate of complete remission (25). CAR-T cells bypass the traditional T cell’s MHC-dependent recognition and directly target the CD19 antigen, effectively addressing the issue of antigen downregulation in lymphoma (162).

However, over time, some patients develop resistance to CAR-T therapy, usually due to tumor cells escaping immune surveillance by losing CD19 antigen expression or increasing the expression of immune checkpoint molecules (163–165). To address this issue, researchers are developing bispecific CAR-T cells that target multiple antigens simultaneously, reducing the risk of immune escape (166, 167).

### 5.5 ACT in other solid tumors

In addition to melanoma, lung cancer, breast cancer, and lymphoma, the application of adoptive immunotherapy in other solid tumors is also continuously expanding. For instance, in clinical trials for prostate cancer (23), hepatocellular carcinoma (168), and gastric cancer (169), ACT therapy has shown potential to improve immune escape mechanisms. These tumors often evade T cell attacks through metabolic suppression and immune checkpoint escape (97–99). By combining immune checkpoint inhibitors with ACT treatment, the survival and response rates of patients with these solid tumors have been significantly improved (156, 160, 170).

## 6 Challenges and prospects of ACT

### 6.1 Current challenges

ACT has shown great promise in cancer treatment but faces multiple challenges. First, the high cost and complexity of the treatment limit its widespread application. ACT requires the *in vitro* expansion and genetic modification of a patient’s T cells, a process that is time-consuming and expensive (10). Second, off-target effects are one of the major safety risks, especially in CAR-T therapy, where T cells may attack healthy tissues, leading to severe side effects (12). Tumor heterogeneity and antigen loss also make some patients unresponsive to treatment, particularly in solid tumors where the immunosuppressive TME weakens the durable action of T cells (63). T cells may become functionally inactivated in the TME, making it difficult to efficiently infiltrate and continuously kill tumors (6). Furthermore, the challenges include the inactivation of immune cells, restricted localization, and diminished efficacy. To tackle these issues, future research must focus on optimizing T cell modification strategies to enhance both efficacy and targeting precision.

### 6.2 Prospects for new technologies

The future prospects for ACT are broad, especially driven by new technologies. Gene-editing technologies, such as CRISPR-Cas9, are significantly changing the outlook for ACT applications. With CRISPR technology, scientists can precisely edit the genes of T cells, removing inhibitory signal molecules to enhance their anti-tumor activity (171). For instance, by knocking out PD-1 or other immune checkpoint molecules on the surface of T cells, it is possible to prevent tumors from evading immune surveillance through immune checkpoint pathways (10). Furthermore, CRISPR technology can integrate novel antigen receptors, thereby enhancing T cells’ tumor recognition capabilities (63, 171).

In the future, the combined application of ACT with other treatments will further improve therapeutic effect. The combination of ACT with immune checkpoint inhibitors (such as PD-1/PD-L1 inhibitors) has already shown promising effects in multiple clinical trials (6, 156, 160, 170). Moreover, Combining ACT with targeted therapies (such as BRAF inhibitors) also brings new hope for patients with various solid tumors (12, 172). This multi-pronged treatment strategy not only enhances the efficacy of T cells but also improves the immunosuppressive state of TME, reducing the tumor’s immune escape (80). Future research directions involve continuously optimizing gene-editing technologies and combination therapies to further enhance the anti-tumor capabilities of ACT and reduce side effects (2).

### 6.3 Personalized ACT therapy

Personalized ACT aims to achieve precision treatment for each patient by detecting tumor-specific antigens and designing personalized treatments. Tumor-specific antigens, such as neoantigens, are proteins expressed in tumor cells due to mutations or abnormal gene expression (70), and can be recognized by T cells. With next-generation sequencing technology, it is possible to quickly screen and identify specific antigens for each patient (10, 171). Using this data, researchers can design customized T cell treatment plans, such as TCR-T or CAR-T therapies, to target tumor cells expressing these antigens (63). Personalized design also involves genetic modification of T cells to enhance their survival and killing efficiency in the TME (173–175). This tailored approach not only improves therapeutic effect but also reduces off-target effects and side effects (12). As precision medicine advances, personalized ACT will become a mainstream direction in cancer treatment.

## 7 Conclusion

ACT has demonstrated significant potential in combating tumor immune evasion by enhancing the immune system’s anti-tumor capabilities. This article has detailed how ACT effectively counters the complex strategies of tumor escape through various mechanisms, including enhancing T cell activity, improving the TME, overcoming immune checkpoint inhibition, and metabolic suppression. Furthermore, the article has explored the application of emerging technologies such as CRISPR gene editing, showing the future direction of personalized ACT treatment, especially its broad prospects in combination with immune checkpoint inhibitors and targeted therapies. Although ACT therapy faces challenges such as high costs, off-target effects, and tumor heterogeneity, with the application of new technologies and continuous optimization of treatment plans, ACT is expected to become a key strategy in conquering cancer.
